# Addressing colorectal cancer screening gaps in Uruguay: a health literacy perspective

**DOI:** 10.1007/s10552-026-02131-6

**Published:** 2026-02-06

**Authors:** Lydia P. Buki, Micaela Reich, John M. Abbamonte, Robert K. Sommer, Selva Sanabia, Dolores Larrosa, Bibiana Sologaistoa, Mercedes Blanco

**Affiliations:** 1 Department of Educational and Psychological Studies, University of Miami, 5202 University Drive, Coral Gables, FL 33146 USA; 2 Departamento de Bienestar y Salud, Universidad Católica del Uruguay, Montevideo, Uruguay; 3 Wall Township, NJ USA; 4 Area de Educación Poblacional, Comisión Honoraria de Lucha Contra el Cáncer, Montevideo, Uruguay

**Keywords:** Cancer control, Colorectal neoplasms, Early detection, Latin America, Psycho-oncology, International

## Abstract

**Purpose:**

Uruguay has the highest colorectal cancer (CRC) mortality rate in the world, given the high prevalence of risk factors and low screening rates. Despite national guidelines recommending regular FIT screening between the ages of 50 and 74, less than half of the eligible population has obtained the test. Clearly, efforts are needed to increase screening rates. Little is known, however, about factors that promote FIT uptake among Uruguayans. To address this research gap, a theoretical analysis was conducted based on the health literacy model, to understand the relative contribution of individual and organizational health literacy variables in screening behaviors.

**Method:**

Through community-based outreach, a national sample of 398 Uruguayan women and men was recruited. Participants had never been diagnosed with CRC and were 50 to 74 years of age, consistent with national screening guidelines.

**Results:**

Analyses showed that when examining individual health literacy, knowledge of cancer more generally, and of CRC in particular, had the largest predictive value. However, when adding organizational health literacy variables to the model, recommendation by a health care provider yielded odds of screening 210 times higher.

**Conclusion:**

The dramatic effect of a provider’s recommendation has important implications for future theory building, research, and practice. Additionally, this study supports the use of a health literacy conceptualization to understand predictors of cancer screening in Uruguay.

## Introduction

Colorectal cancer (CRC) is the third most common cancer worldwide [1]. Its mortality rate is the second highest of all cancers, with a concerning upward trend in individuals under the age of 50 [2, 3]. In Uruguay, adoption of the Western diet and lifestyle behaviors have placed its residents at higher risk [4]. In fact, CRC incidence rates in Uruguay (2016–2020) are among the highest in the Americas [5], with mortality rates in 2021 being the highest in the world [6]. Therefore, there is a great need for early screening as well as information to guide prevention and control efforts. Across cancer types, CRC incidence and mortality rates in Uruguay are the second-highest for women (24.12 and 11.20 per 100,000, respectively) and third-highest for men (36.63 and 18.58 per 100,000, respectively) [7], with incidence rates increasing for Uruguayans under the age of 50, ages 40–49 [8]. Most concerning are projections suggesting that approximately 4% of the population will be diagnosed with CRC in their lifetime, while the expected overall 5-year survival rate is modest, at 55% (not including cancer in situ; otherwise can vary by stage at diagnosis) [9]. These statistics are amenable to change because CRC risk factors are largely modifiable and routine screening can detect precancerous polyps and cancer in early stages [2, 10].

The Uruguayan public health authority, *Ministerio de Salud Pública* [11], issued recommendations for individuals ages 50–74 without risk factors to obtain a Fecal Immunochemical Test (FIT) every 2 years. A facilitative factor for screening in Uruguay is that it has a healthcare safety net that typically decreases the cost of the exam significantly, expanding access [12, 13]. Yet, in 2013, only 36% of men and 46% of women 50 to 64 years of age had obtained the test at least once [14]. Thus, it is critical to further understand facilitative factors and barriers to screening in this high-risk population.

Advances in screening place complex cognitive, affective, and behavioral demands on individuals: they need to (a) have information about the cancer type, (b) understand their personal risk, (c) be aware of ways to screen for the cancer, (d) know the current screening guidelines, (e) know the process for obtaining screenings, and (f) follow through with the process [15]. To understand and conceptualize factors that influence screening behaviors and identify foci for intervention, the health literacy model, which has been used in previous early detection studies [4, 15, 16], provides a suitable framework. According to this framework, there are two types of health literacy: individual [17] and organizational [18]. *Individual health literacy* refers to “the degree to which individuals have the capacity to obtain, process, and understand basic health information and services needed to make appropriate health decisions” [17]. This complex construct includes cultural schema, print and oral literacy, and numeracy [19, 20]. Specifically, *cultural schema* refers to the “filter” through which health information is obtained, processed, and understood, and includes knowledge, beliefs, attitudes, and emotions relevant to a behavioral outcome (e.g., cancer screening) [16, 19].

Indeed, individuals with adequate health literacy obtain CRC screenings at higher rates than those with lower levels [21–23]. Aspects of cultural schema that correlate with low CRC screening rates include having limited knowledge about CRC and the purpose of screening exams [24–26], lacking confidence in the healthcare system, exhibiting mistrust of individual providers, holding negative beliefs and attitudes about healthcare encounters with providers, and holding negative beliefs and attitudes about cancer [25, 26].

Organizational health literacy also influences screening rates. Service providers, community-based organizations, and other health systems need to be responsive to the population’s literacy needs by making “health information and resources available and accessible to people according to [their] health literacy strengths and limitations” [27]. Organizational correlates of low CRC screening include cost, being uninsured, and lacking a screening recommendation from a medical professional [25, 28, 29]. Conversely, facilitators of CRC screening include exposure to national health promotion campaigns, higher SES [30], and receiving a screening recommendation from a healthcare professional [28, 31, 32].

Despite the need to increase CRC screening rates in Uruguay, extant research has primarily focused on risk factors such as diet and genetic profiles [33, 34]. To our knowledge, no study has sought to develop a theoretically and empirically based model of CRC screening behavior to guide interventions at a public health level. To fill this gap in the literature, consistent with the health literacy model, we examined contributing factors at the individual and organizational levels. At the individual level, we focused on cultural schema because even when lowering the literacy level of print materials, cultural factors may act as powerful barriers to screening. Given the study’s theoretical base, the findings have the potential to guide screening efforts in the future, optimizing use of funds for prevention and ultimately lowering the country’s CRC cancer burden.

## Materials and methods

### Participants

Participants were born in Uruguay, had never been diagnosed with CRC, and were 50 to 74 years of age, consistent with national screening guidelines. To ensure a diversity of viewpoints and experiences, participants were recruited nationally across five *departamentos* (similar to U.S. states) representing urban and rural contexts across Central, Eastern, Northern, and Southern areas. These data represent a secondary analysis of a national Uruguayan dataset collected in 2018 used to perform a psychometric validation of the Colorectal Cancer Literacy Scale–Uruguay (CCLS–U) [35]. A subset of the sample without missing data on key study variables (*n* = 398) allowed us to examine theoretical associations across key health literacy variables. In this article, we present original findings not previously published.

On average, participants were 60.3 years of age (*SD* = 6.9, *Mdn* = 59.0). The sample was evenly split between genders, with 202 (50.8%) identifying as women and the remainder as men. Almost two-thirds were married or cohabiting (*n* = 256; 64.3%). Formal educational attainment ranged from 0 to 29 years (*M* = 11.2, *SD* = 4.4, *Mdn* = 11.0). Over a third (*n* = 145; 36.4%) were no longer in the workforce. The average annual household income was 599,700.72 pesos (approximately $17,382.63 USD; *SD* = 671,833.20 pesos, *Mdn* = 480,000.00 pesos; *n* = 395). Participants had access to healthcare: 248 (62.3%) were members of a *mutualista* (akin to an HMO) and 119 (29.9%) were members of the public safety net system, *ASSE*.

### Procedure

Community gatekeepers on staff at the *Comisión Honoraria de Lucha Contra el Cáncer*, a national cancer education and advocacy organization, recruited participants in community settings at each *departamento*. They set out to recruit 40 women and 40 men in each location, using their knowledge of the community and local organizations to (a) identify potential participants, (b) attend community events and make announcements about the study, and (c) identify additional participants through snowball sampling. Institutional Review Board approval was obtained both from the first author’s institution in the United States and from the Uruguayan National Cancer Institute (INCA). After ascertaining inclusion and exclusion criteria and obtaining oral consent from participants, gatekeepers verbally administered the scale, entering responses electronically on a password-protected tablet. To ensure anonymity, no identifiable information was collected. On average, it took 20 min to gather data from each participant. Participants did not receive compensation for participating in the study.

### Measures

#### Background questionnaire

The questionnaire included 10 demographic questions to gather information from participants including age, gender, level of formal education, marital status, type of health insurance, household income, and occupational status. Additionally, it included 13 questions related to CRC. For instance, items asked whether participants (a) had ever screened, including reasons for screening or for not obtaining the exam; (b) had a family history of CRC; (c) had ever received a recommendation to obtain a FIT from a healthcare provider; (d) had ever attended a workshop on the importance of cancer screening (more broadly); and (e) had ever received information about the importance of CRC screening (more specifically), whether from a healthcare provider, by attending a community health program, and/or through a national campaign. Any such exposure to the importance of CRC screening was coded as having been exposed to information from a formal source.

#### CCLM–U [35]

This scale, which measures cultural schema related to CRC (a component of CRC health literacy), has a total of 44 items across three subscales: Subscale I, Disposition Toward Cancer Prevention; Subscale II, Attitudes, Beliefs, and Emotions About Cancer; and Subscale III, Knowledge About CRC. The measure, which was validated with a Uruguayan sample, showed adequate Expected a Posteriori reliabilities and good initial discriminant validity. Sample items include “If I noticed a symptom of CRC, I would go to the doctor to get it checked” (Subscale I) and “A symptom of colorectal cancer is blood in the stool” (Subscale III). Subscales I and II are measured on a Likert-type, 5-point scale with total scores ranging from 15 to 75 for Subscale I, and 9 to 45 for Subscale II. Answers for Subscale III items include “Yes,” “No,” and “I do not know” (the latter was considered an incorrect answer). Respondents score one point for each correct answer. The range of possible scores for this subscale is 0 to 20. Across subscales, higher ratings indicate more favorable responses.

## Results

### Preliminary descriptive information

Although all participants were within the age range for recommended screening, 14% (*n* = 56) were unsure they were due for screening or thought they were not due. Of those who reported being within the recommended age range for screening, only 22% (*n* = 88) correctly noted they should obtain the test every 2 years.

Descriptive statistics were calculated for the subscales: Disposition Toward Cancer Prevention (*M* = 50.5, *Mdn* = 51.0, *SD* = 4.8); Attitudes, Beliefs, and Emotions about Cancer (*M* = 29.9, *Mdn* = 30.0; *SD* = 4.7); and Knowledge about CRC (*M* = 14.6, *Mdn* = 15.0, *SD* = 3.2). On average, participants had moderate scores for Disposition Toward Prevention and Attitudes, Beliefs, and Emotions About Cancer. Yet, Knowledge scores indicate that, on average, participants answered 75% of items correctly. As shown in Table 1, over half of participants (61.3%; *n* = 244) reported no exposure to CRC information from a formal source, and three-fourths (75.1%; *n* = 299) reported not having attended a workshop on the topic of cancer screening. In contrast, seven out of every 10 participants (70.1%; *n* = 279) reported receiving a recommendation to obtain a FIT from a healthcare provider. Over a fifth of participants (21.6%; *n* = 86) reported neither being exposed to information nor receiving a screening recommendation from a medical professional.

**Table 1:** Workshop attendance, exposure to information, and recommendation as a function of FIT status

Variable	FIT status		*p*
	No	Yes	
*Attended workshop about cancer screening*			.174
No	101 (33.8%)	198 (66.2%)	
Yes	26 (26.3%)	73 (73.7%)	
*Exposure to information from formal source*			.015
No	89 (36.5%)	155 (63.5%)	
Yes	38 (24.7%)	116 (75.3%)	
*Health professional FIT recommendation*			< .001
No	110 (92.4%)	9 (7.6%)	
Yes	17 (6.1%)	262 (93.9%)	

*p*-values based on Fisher’s exact tests

Overall, almost seven out of every 10 participants (68.1%; *n* = 271) reported obtaining at least one FIT; almost four out of every 10 screened participants (38.7%; *n* = 154) reported obtaining more than one FIT, with a small percentage (1%; *n* = 4) unsure whether they were rescreened. Positive associations were observed between having ever been screened and (a) exposure to information from a formal source (*p* = .015) and (b) receiving a screening recommendation (*p* < .001). No association was found between exposure to information about cancer screening at a workshop and FIT uptake.

Based on available data about family cancer history (*n* = 385), a significant association was found between receiving a screening recommendation and having a family history of CRC (*p* = .009). Among individuals with a family history (*n* = 71), 83.1% (*n* = 59) received a screening recommendation compared to 67.5% (*n* = 212) among those not reporting a family history (*n* = 314).

With respect to rescreening, participants ages 53 and older (*n* = 342), who would have been expected to have obtained at least two screenings, were examined separately. Among this subgroup, of the 240 participants who reported obtaining at least one screening (70.2%), only 142 (59.2%) had rescreened. This means that over 40% were not adherent at the time of their first rescreening timepoint.

### CCLS–U subscale correlations

All intercorrelations were statistically significant: Disposition Toward Cancer Prevention was positively correlated with Attitudes, Beliefs, and Emotions about Cancer (*r* = .54, *p* < .001) as well as with Knowledge About CRC (*r* = .43, *p* < .001). Additionally, Attitudes, Beliefs, and Emotions about Cancer was positively correlated with Knowledge about CRC (*r* = .18, *p* < .001), although the strength of this association was lower than the other intercorrelations.

### Logistic regressions

#### Baseline logistic regression

To examine the contribution of cultural schema to FIT uptake, the three CCLS–U subscales were entered into a generalized linear model with a logit link, binomial family, and robust standard errors (see Table 2), yielding a statistically significant model, *χ*^2^(3) = 21.1; *p* < .001; LL = −228.2; AIC = 464.3; BIC = 480.3. Within the model, both Disposition Toward Cancer Prevention (*b* = 0.06, *SE* = 0.03; *p* = .034) and Knowledge About CRC (*b* = 0.1, *SE* = 0.04; *p* = .006) were significant predictors of FIT uptake. Exponentiating the coefficients revealed that, on average, each 1-point increase in Disposition increased the odds of receiving a FIT by 6.2% while controlling for other variables in the model. In turn, each 1-point increase in Knowledge, on average, was associated with an increase in the odds of receiving a FIT by 10.6%.

**Table 2:** Baseline logistic regression predicting FIT uptake

Variable	*b*	*SE*	*χ*^2^	*p*
Intercept	−3.34	1.13	8.73	.003
DTP	0.06	0.03	4.49	.034
ABEC	−0.01	0.03	0.19	.662
KCRC	0.1	0.04	7.61	.006

Logistic regression using a binomial family with logit link and robust standard errors. Having obtained a FIT was treated as the response category. *DTP* Disposition Toward Prevention; *ABEC* Attitudes, Beliefs, and Emotions About Cancer; *KCRC* Knowledge About CRC

#### Full logistic regression model

Next, a logistic regression was performed to evaluate the contribution of organizational literacy to FIT uptake over and beyond that of cultural schema (see Table 3). Two variables were added to the model: (a) number of exposures to information about CRC screening, and (b) receipt of a medical professional’s FIT recommendation. Again, this model was statistically significant, *χ*^2^(5) = 310.8; *p* < .001; LL = −91.7; AIC = 195.5; BIC = 219.4. A log-likelihood test indicated that these additions significantly improved the model overall (2ΔLL = 272.86, ΔDF = 2; *p* < .001). Within the model, only a medical recommendation (*b* = 5.3, *SE* = 0.43; *p* < .001) significantly predicted FIT uptake. Exponentiating the coefficients revealed that, on average, receiving a FIT recommendation from a medical professional yielded odds 210 times higher while controlling for the variables previously entered in the model.

**Table 3:** Logistic regression predicting FIT uptake with organizational health literacy variables

Variable	*b*	*SE*	*χ*^2^	*p*
Intercept	−4.19	2.19	3.67	.055
DTP	−0.02	0.06	0.09	.758
ABEC	0.03	0.05	0.3	.587
KCRC	0.13	0.07	3.44	.064
Health professional FIT recommendation	5.35	0.43	152.31	< .001
‍Exposure to information	−0.39	0.26	2.25	.133

Logistic regression using a binomial family with logit link and robust standard errors. Having obtained a FIT was treated as the response category. Reference category for recommendation was treated as not having had a FIT recommendation. *DTP* Disposition Toward Prevention; *ABEC* Attitudes, Beliefs, and Emotions About Cancer; *KCRC* Knowledge About CRC

Despite these clear findings, we sought to deepen our understanding by examining the reasons reported by participants for not obtaining a FIT. Consistent with the aforementioned results, among individuals who had never screened (*n* = 127), the top reasons were not receiving a recommendation for the exam (47.2%), feeling fine (26.0%), and not understanding the purpose of the exam (16.5%). Specifically, among the subgroup of participants who had not screened and had not received a healthcare provider recommendation (*n* = 115), not having a recommendation was the most frequently endorsed barrier (50.0%). Among the remaining participants who had received a recommendation but had not screened (*n* = 12), endorsed barriers included feeling fine (25.0%), lack of interest in getting the exam (25.0%), the perception that the exam was not necessary at their age (16.7%), and fear of diagnosis (16.7%).

## Discussion

To our knowledge, this is the first attempt to develop an empirically based model of individual and organizational health literacy factors that influence FIT uptake. Results from a national community-based sample in Uruguay show that a construct related to organizational health literacy (i.e., receiving a screening recommendation from a health professional) had a much stronger association with screening than constructs related to individual health literacy (i.e., cultural schema). After controlling for cultural schema, receiving a recommendation from a healthcare provider resulted in odds of screening 210 times higher, a dramatically larger effect than we have seen documented in the literature [32]. Thus, our data contribute to the growing literature supporting the positive association between health literacy and CRC screening [36].

Given that the process of obtaining the exam is triggered when a healthcare provider makes a referral, receiving a recommendation from a healthcare provider would be expected to contribute to screening behavior. The strong effect is likely heightened by cultural factors: being part of a hierarchical culture, Uruguayans are likely to ascribe much power to medical providers, trusting their expertise and taking their recommendations quite seriously. Also, conversations about FIT uptake will take place mainly when a provider broaches the subject.

An individual-level facilitative factor to screening in the baseline model was disposition toward cancer prevention, consistent with previous research supporting the notion that those who perceive that screening will be beneficial are more likely to engage in CRC screening [37]. A second individual-level facilitative factor was knowledge, which yielded a larger effect. Although this effect was subsequently overshadowed by organizational factors in the full model, our results are similar those in the literature, suggesting the important role of knowledge on screening uptake [24–26]. Consistent with this, barriers to screening endorsed by participants largely related to lack of knowledge about (a) CRC symptoms (e.g., not screening because they were feeling fine), (b) the FIT (e.g., not understanding the purpose of the exam), or (c) screening guidelines (e.g., perceiving the exam was not necessary at their age). Thus, there is a need to educate the community about these topics.

Notably, all participants were of screening age, yet over 1 in 5 reported both (a) not receiving a screening recommendation, and (b) no exposure to information about the importance of early CRC detection. Greater efforts are needed to reach those who may otherwise lack access to information, whether through health promotion campaigns or by disseminating information through medical systems and health providers. This is especially important because when the FIT results are abnormal, all Uruguayans have access to a colonoscopy and, if needed, cancer treatment, whether through private insurance or the national health care safety net.

### Implications for practice and future research

Further research is warranted to develop and evaluate programs designed to disseminate evidence-based information. There is a national CRC screening promotion campaign underway in Uruguay that provides information about national screening guidelines, the nature of CRC, and healthy eating [38]. We recommend expanding and evaluating current practices. Based on our findings, topics to cover include CRC’s asymptomatic nature, the need to screen in the absence of symptoms, the age at which screening is necessary, and the FIT’s purpose, among others (see Table 4). In addition, tailoring would be necessary to address beliefs, attitudes, and emotions that may act as barriers to screening, such as the belief that lack of symptoms is associated with colorectal health, the attitude that screening is not a priority, and fear of diagnosis. For instance, for those who fear the FIT may show cancer, it would be important to provide testimony from individuals who survived due to early detection. Given that less than half of participants over the age of 53 had rescreened, rescreening intervals should be presented as well.

**Table 4:** Reasons for not screening and recommendations for health education

Reasons	Recommendations
Not receiving a recommendation for the exam	• Explain the importance of early detection • Provide information about screening guidelines • Present data on low screening rates • Empower individuals to ask for a referral for the exam
Feeling fine	• Explain the asymptomatic nature of polyp development and early-stage CRC • Articulate the need for screening and rescreening even when feeling fine
Not understanding the purpose of the exam	• Explain the process and goal of the exam
Lack of interest in getting the exam	• Provide information about low screening rates • Discuss prognoses as a function of cancer stage • Provide information about screening guidelines and need to rescreen
Perception that they did not need to obtain the exam at their age	• Provide information about screening guidelines • Encourage close medical surveillance when having known risk factors
Fear of being diagnosed	• Explain link between early detection and prognosis • Provide role models of people who survived CRC due to early detection • Promote knowledge of risk factors

We also note that, importantly, 7 out of every 10 participants reported receiving a screening recommendation. Moreover, participants at higher risk due to a family history of CRC were more likely to receive a recommendation than those without this history. Overall, health care providers seem knowledgeable about their patients’ CRC family history, which may require screening at earlier ages and shorter intervals. Consistent with these findings, we note that there is an optional course offered at UdelaR university’s medical school in Montevideo, titled “*Hacia un mejor control de cáncer*” [“Toward Enhanced Cancer Control”] whereby pre-medical students receive training on cancer prevention generally, and CRC prevention specifically. The course includes a 3-h unit on early detection of CRC and exposes students to relevant theory and practice. This is a very important initiative in light of our findings. Research is warranted to assess the effectiveness of this program as well as that of other national initiatives designed to promote early CRC detection. Funds should be designated for research and strategic planning to develop comprehensive strategies aimed to increase baseline screening and rescreening rates.

### Contributions and limitations of the study

Despite the fact that CRC represents a significant public health issue in Uruguay, this is the first empirical effort to examine individual and organizational predictors of screening in a national community-based sample. Findings suggest the critical role of organizational and individual health literacy in promoting FIT uptake. Yet, the study has limitations. First, data were self-reported by participants, which may have introduced error in measurement, should reports be inaccurate. It is possible that factors such as social desirability may have biased the results, as data were collected in person and verbally, rather than participants entering the data themselves in an electronic medium. We felt it was important for the community gatekeepers to ask the questions and enter the data themselves given that some participants were likely to have difficulty entering the data on their own due to low print and technical literacy. Additionally, we asked participants whether they had received a recommendation to obtain the screening, but it is unclear whether the recommendation was always coupled with a referral for screening. Although this is likely to have been the case, we recommend assessing receipt of a screening referral rather than a screening recommendation in future research.

With regard to external validity, special care should be exercised when generalizing to countries without a public health safety net. It is unclear whether factors such as receiving a screening recommendation or having exposure to information may influence screening similarly in countries with more limited access to health care. Also, findings may not be generalizable to countries where healthcare providers are not ascribed such high regard. The relatively Western culture and strong economy of Uruguay, compared to less Westernized countries in the Americas, may also limit the findings’ generalizability.

In contrast, the diversity exhibited by participants in our sample as well as the representation of various regions across the country suggest that our findings possess strong generalizability within Uruguay. Also, based on similarities between Uruguay and other countries in South America such as Argentina, it is possible that our findings would have strong external validity across the neighboring region.

Overall, results suggest a need to combine knowledge with access. Typically, access is conceptualized as having health insurance or a usual source of care. In a country with a health care safety net, receiving a recommendation from a healthcare provider greatly facilitated access to screenings. Thus, the study contributes to the ongoing dialogue related to the relative influence of individual and organizational factors on health promotion behaviors. Our data showed that a provider’s screening recommendation overshadowed the influence of individual cultural barriers including beliefs, attitudes, emotions, and knowledge.

## Conclusions

This study confirms that a health literacy conceptualization yields valuable insights regarding predictors of cancer screening in Uruguay. Moreover, findings suggest that although individual literacy factors are important in understanding screening behavior, the effects are overshadowed by organizational health literacy factors. Noteworthy implications emerged for future theory building, research, and practice.

## Data Availability

Data available on request due to privacy/ethical restrictions.

## Declarations

### Research involving human and animal participants

The study was approved by the University of Miami Human Subjects Research Office and the *Instituto Nacional del Cáncer* (INCA) in Uruguay, and was conducted in accordance with the 1964 Declaration of Helsinki as well as local legislation and institutional requirements.
